# Liver Disease as a Potential Risk Factor for Colorectal Cancer: A Community Hospital Experience

**DOI:** 10.7759/cureus.62400

**Published:** 2024-06-14

**Authors:** Daniel Aillaud-De-Uriarte, Luis A Hernandez-Flores, Philip N Zachariah, Ria Bhatia, Hairé Manzano-Cortés, Diego Marines-Copado

**Affiliations:** 1 Center for Bioethics, Harvard Medical School, Boston, USA; 2 Division of Colon and Rectal Surgery, Houston Methodist Willowbrook Hospital, Houston, USA; 3 Gastroenterology, Drexel University College of Medicine, Philadelphia, USA; 4 Epidemiology and Biostatistics, The University of Texas at Austin, Austin, USA; 5 Gastroenterology and Hepatology, Hospital Angeles Puebla, Puebla, MEX

**Keywords:** cancer, colonoscopy, masld, liver disease, colorectal cancer

## Abstract

Background: Liver disease (LD) is a common pathology worldwide. Many patients remain asymptomatic and undiagnosed. Colorectal cancer (CRC) is a prevalent neoplasm and a leading cause of cancer-related deaths globally. Multiple studies suggest that inflammation in the liver could drive the initiation of colorectal cancer.

Methods: This five-year (2018-2022) case-control study included 274 patients diagnosed with CRC and adenomas at a community hospital in Houston, Texas. Each patient’s medical record was reviewed for pre-existing LD, including steatosis, cirrhosis, primary biliary cirrhosis, and Hepatitis B and C infections. This study aims to investigate the association between LD and CRC risk and assess differences by gender, race, and ethnicity. The study cohort comprised 124 (45.3%) women and 150 (54.7%) men. Data were compared and analyzed using a Chi-squared test for independence and binomial logistic regression. A p-value of < 0.05 was considered statistically significant.

Results: Patients with LD had a two-fold increase in the odds of developing CRC compared to those without LD, in both univariate and multivariate analyses (OR 2.13 {95% CI 1.30-3.49}, p = 0.003 / OR 2.30 {95% CI 1.37-3.87}, p = 0.002, respectively). The chi-square test revealed that the association between CRC and LD was stronger in women than in men (p = 0.018 and p = 0.056, respectively).

Conclusion: Our study establishes a positive correlation between LD and CRC development, suggesting LD is a potential risk factor for CRC, particularly in women. Future research directions include exploring the underlying mechanisms of this association, evaluating the utility of early CRC screening in individuals with LD, and assessing the impact of interventions targeting LD on CRC incidence and mortality.

## Introduction

An abstract containing information on this paper was previously presented at Digestive Disease Week (DDW 2024), Washington, D.C., as "Tu1150 Liver Disease as A Risk Factor for Early Colorectal Screening" (https://doi.org/10.1016/S0016-5085(24)03338-9).

Liver disease (LD) is estimated to affect 1.5 billion people worldwide, many of whom remain asymptomatic and undiagnosed, accounting for over two million deaths annually and being the eleventh-leading cause of death [1]. Metabolic dysfunction-associated steatotic liver disease (MASLD) is the most common cause of chronic LD, with its prevalence having increased from 22% to 37% [1,2]. This increase parallels the rise in obesity and type 2 diabetes worldwide [2]. Within North and South America, higher prevalence rates have been reported in Mexico and Brazil, whereas lower prevalence rates have been seen in Canada and Argentina, suggesting that MASLD is also influenced by ethnicity [3]. Metabolic syndrome has become a global pandemic, with its prevalence increasing due to an unhealthy lifestyle that includes a lack of physical activity, poor diet, smoking, and increased alcohol consumption. In the United States, the prevalence of LD increased from 36.2% in 1999-2000 to 47.3% in 2017-2018 [2,3]. 

Just as LD affects a large portion of the population, colorectal cancer (CRC) is one of the most diagnosed cancers worldwide, accounting for approximately 10% of all annually diagnosed cancers and cancer-related deaths [4]. It has well-established risk factors, including hereditary syndromes (Lynch syndrome, Gardner syndrome, familial adenomatous polyposis). However, it has been shown that 75-95% of CRC cases occur in individuals with little or no genetic risk, prompting the evaluation of other risk factors [5]. Male sex, increasing age, and inflammatory bowel disease have all been shown to be non-modifiable risk factors, whereas several modifiable lifestyle risk factors such as smoking, excessive alcohol intake, and obesity have been identified [6]. 

CRC is mostly diagnosed in adults between 50 and 74 years old, accounting for 60.4% of CRC patients [7,8]. Colonoscopies are recommended by the US Preventive Services Task Force (USPSTF) to be performed by the age of 45 in non-high-risk patients [9]. Unfortunately, up to 10% of CRC cases are diagnosed in individuals under 50. North America has the highest mortality rates globally among males younger than 50 years old [7,8]. It is predicted that by 2040, 3.2 million cases will occur. Elevated CRC incidence reflects the increase in overweight levels, decrease in physical activity, and increase in alcohol and smoking consumption [10]. CRC's elevated mortality demonstrates insufficient optimal screening and treatment services [7]. 

This study aims to assess LD as a potential risk factor for CRC and to explore their relationship. Our hypothesis is that individuals with LD, particularly MASLD, have an increased risk of developing CRC. By investigating this association, we seek to contribute to the understanding of CRC's etiology and identify new risk factors that may guide prevention and screening strategies. This research builds on existing studies that have identified common modifiable risk factors for both LD and CRC, such as obesity and alcohol consumption. However, there is a need for more specific investigation into the direct relationship between LD and CRC.

## Materials and methods

A five-year case-control study was performed, which included 274 patients admitted to the Houston Methodist Willowbrook Hospital, a community hospital in Houston, Texas, between January 2018 and December 2022.

The chosen study period allowed for a comprehensive collection of cases and controls, ensuring sufficient data for meaningful analysis. This community hospital setting was selected due to its diverse patient population, providing a representative sample of the local demographics and healthcare patterns.

Data were collected from all the patients diagnosed with CRC or colorectal adenomas. The diagnoses of CRC and colorectal polyps were confirmed by colonoscopy and histopathology in 100% of cases. Each patient's electronic medical record (EMR) was screened for pre-existing LD.

Inclusion criteria were patients with confirmed diagnoses of CRC and adenomas. Exclusion criteria included patients with missing critical information (e.g., colonoscopy report), history of Lynch syndrome, familial adenomatous polyposis, inflammatory bowel disease (IBD), age below 18 years, and pregnant women. We also excluded patients with other types of chronic liver diseases not directly related to metabolic dysfunction (e.g., autoimmune hepatitis, Wilson’s disease). A total of 274 patients were consecutively enrolled to obtain a representative sample.

The clinical and demographic variables included age, sex, race, body mass index (BMI), history of type 2 diabetes, dyslipidemia, alcohol and tobacco consumption, pre-existing LD (including steatosis, cirrhosis, primary biliary cirrhosis, hepatitis B virus {HBV}, and hepatitis C virus {HCV}), CRC, adenoma location, and metastasis if present. Glycosylated hemoglobin (HbA1c), high-density lipoprotein (HDL), low-density lipoprotein (LDL), triglycerides (TG), alanine aminotransferase (ALT), aspartate aminotransferase (AST), and carcinoembryonic antigen (CEA) values were recorded in the database. Additionally, the patient’s colorectal surgical procedure, if required, was recorded, including a pathology report. The patient's history of cancer and adenoma recurrence was also registered.

To handle missing data, multiple imputation methods were applied where feasible, and sensitivity analyses were performed to assess the impact of missing data on the results. Selection bias was minimized by consecutively enrolling patients who met the inclusion criteria and by using standardized data collection methods. Information bias was addressed through rigorous validation of diagnoses using colonoscopy and histopathology reports.

Statistical analysis was performed with IBM SPSS Statistics for Windows, Version 27.0 (IBM Corp., Armonk, NY, USA). Categorical variables were expressed as frequencies and percentages, while continuous variables were expressed as means and standard deviations or medians and ranges, depending on the distribution. The Chi-square test was used for comparing categorical variables due to its suitability for analyzing the association between categorical data. Binomial logistic regression was employed to identify potential predictors and control for confounding variables. Continuous variables were compared using the Student’s T-test or the Mann-Whitney U test based on the normality of the distribution, assessed using the Shapiro-Wilk test. A p-value of < 0.05 was considered statistically significant.

Using risk factors previously associated with the literature, we performed an exploratory analysis to identify the associations between CRC and LD. The rationale for choosing these statistical tests was based on the need to compare proportions between groups and to control for multiple confounding factors simultaneously.

This case-control study was approved by the Research Institute Committee, Houston Methodist Willowbrook Hospital, with approval number PRO00037701 and conducted in accordance with the ethical standards of the Declaration of Helsinki.

## Results

From the analysis of 276 patients diagnosed with CRC and adenomas, two patients were excluded due to a diagnosis of Lynch syndrome, resulting in a final population of 274 (n = 274). The sample included 124 women (45.3%) and 150 men (54.7%), with a mean age of 63 (±12) years. Regarding ethnicity, there were 51 Hispanic or Latino patients (19%) and 223 non-Hispanic or Latino patients (81%). In terms of race, there were 227 Caucasian (83%), 33 Black (12%), nine Asian (3%), and five Native American (1.8%) patients.

Among our population, 151 patients had a CRC diagnosis (55%), while 123 had no CRC (45%). The three main CRC locations were the sigmoid colon (32 patients, 12%), the rectum (31 patients, 11%), and the ascending colon (29 patients, 11%). Pre-existing LD was present in 119 patients (43%), and 162 patients (59%) had adenomas. Adenocarcinoma was the most common histologic type in our CRC population, reported in 135 patients (89%). Regarding comorbidities, 175 patients (64%) were diagnosed with hypertension, 129 with hyperlipidemia (47%), and 71 with type 2 diabetes (26%). Detailed population information is presented in Table 1.

**Table 1:** Patient clinical characteristics and logistic regression. For quantitative variables means ± standard deviations are shown, for qualitative variables frequencies and percentages are shown. The multivariate analysis only included those variables that showed significance in the univariate analysis: liver disease. *Indicates that the p-value is <0.003, representing a statistically significant result. This significance level suggests a strong association between the variables analyzed and the presence of colorectal cancer (CRC) in the studied population. CI: Confidence interval; OR: Odds ratio.

Parameter		Colorectal cancer		p-value	Crude OR (95% CI)	Adjusted OR (95% CI)
		Yes (n=151)	No (n=123)			
Age (years)		64.2 ( ±0.9)	61.7 (± 1.1)	0.093	1.01 (0.99–1.03)	-
Liver disease	Present	78 (52%)	41 (33%)	Univariate 0.003* Multivariate 0.002*	2.13 (1.30–3.49)	2.30 (1.37–3.87)
	Not present	73 (48%)	82 (67%)			
Gender	Female	66 (44%)	58 (47%)	0.951	1.61 (0.56–1.61)	-
	Male	85 (56%)	65 (53%)			
Type 2 diabetes	Present	43 (28%)	28 (23%)	0.283	1.36 (0.78–2.36)	-
	Not present	108 (72%)	95 (77%)			
Hypertension	Present	91 (60%)	84 (68%)	0.192	0.71 (0.43–1.18)	-
	Not present	60 (40%)	39 (32%)			
Hyperlipidemia	Present	66 (44%)	63 (51%)	0.235	0.74 (0.46–1.20)	-
	Not present	84 (56%)	60 (49%)			
Smoking status	Smoker	67 (44%)	65 (53%)	0.151	1.42 (0.88–2.29)	-
	Non-smoker	84 (56%)	58 (47%)			
Alcohol consumption	Yes	77 (51%)	52 (42%)	0.163	0.72 (0.44–1.14)	-
	No	74 (49%)	71 (58%)			

The case group included patients with a confirmed CRC diagnosis (151 patients, 55%). Among them, 78 patients (52%) had documented LD, while the remaining 73 patients (48%) did not. The control group comprised patients without a CRC diagnosis (123 patients, 45%), of which 41 patients (33%) had pre-existing LD. Patients' demographics and other relevant variables were matched between the groups.

Our logistic regression analysis revealed a significant association between LD and CRC in both univariate (Crude OR) and multivariate (Adjusted OR) analyses (Crude OR 2.13 {95% CI 1.30-3.49}, p = 0.003; Adjusted OR 2.30 {95% CI 1.37-3.87}, p = 0.002). After adjusting for confounding variables such as sex, hypertension, smoking, and alcohol consumption, patients with LD had a two-fold increase in the odds of developing CRC compared to those without LD.

The Chi-square test also suggested a significant association between CRC and LD (p = 0.002). When analyzed by gender, we found a significant association for women (p = 0.014) but not for men (p = 0.056), implying that women with LD have a stronger association with CRC. Among different races with LD, the following associations with CRC were observed: Caucasians (p = 0.008), Blacks (p = 0.290), Asians (p = 0.057), and Native Americans (p = 0.709). Non-Hispanics showed a statistically significant association (p = 0.001), while Hispanics did not (p = 0.655) (Table 2). 

**Table 2:** Chi-square association of different races with CRC in patients with LD. *Indicates that the p-value is <0.05, representing a statistically significant result. **Indicates that the p-value is <0.001, representing a strong statistically significant result. CRC: Colorectal cancer, LD: Liver disease.

Race	p-value
Caucasians	0.008*
Black	0.290
Asian	0.057
Native American	0.709
Non-Hispanics	0.001**
Hispanics	0.655

Quantitative variables (HbA1c, HDL, LDL, TG, ALT, AST, and CEA) were not described due to the lack of a true association.

These findings have important clinical implications. The significant association between LD and CRC suggests that patients with LD should be considered for more rigorous CRC screening. The stronger association observed in women and Caucasians indicates that these subgroups may benefit from particularly heightened surveillance.

The absence of a significant association between LD and CRC in patients under 45 years old might be due to the smaller sample size in this subgroup. Additionally, the varying associations observed among different racial groups highlight the need for further investigation into the biological, genetic, and socioeconomic factors that may contribute to these differences. They were not assessed in this study.

Having a previously diagnosed LD did not show a significant association with the risk of developing adenomas (p = 0.114) or metastasis (p = 0.803), suggesting that the relationship between LD and CRC might be more complex and specific to certain pathways of carcinogenesis.

## Discussion

Our investigation spanned a comprehensive five-year period and encompassed a total of 274 patients with a diagnosis of CRC and/or adenomas, wherein 151 patients were diagnosed with CRC, and 45.3% and 54.7% were women and men, respectively. Adenocarcinoma was the most common cancer in our population, reported in 135 patients (49.3%), with the sigmoid colon being the most common location (11.7%). Our logistic regression analysis revealed significant insights into the association between CRC and LD. Patients with LD have a two-fold increase in the odds of developing CRC compared to those without LD (OR 2.30 {95% CI 1.37-3.87}, p = 0.002). Further information can be found in Table 1. Moreover, a comparison between the two groups showed a higher association in women than men, with p-values of 0.014 and 0.056, respectively.

These findings support our hypothesis that LD, is associated with an increased risk of CRC. This is consistent with previous studies, which have demonstrated a link between LD and CRC, highlighting the role of metabolic dysfunction and inflammation in cancer development [10-12]. For example, patients with MASLD exhibit a higher prevalence of precursor lesions and early CRC, independent of other manifestations of insulin resistance [13]. One study found that CRC had a prevalence of 29.3% versus 18% in patients with or without MASLD, respectively, and a higher prevalence of highly differentiated colorectal adenocarcinoma in the MASLD group [14]. Additionally, indices such as the Fatty Liver Index (FLI) have been correlated with an elevated risk of developing esophageal, stomach, and CRC/adenoma, highlighting the predictive value of LD in assessing cancer risk [15,16].

In an era marked by globalization and shifting lifestyle patterns towards sedentarism and high-fat processed diets, the incidence of diseases, including CRC, is escalating, particularly among younger demographics. LD has become a silent pandemic, as MASLD, the most common type of LD, affects at least one in four people worldwide [2,15]. Unfortunately, LD often goes undetected and untreated, exacerbating the risk of long-term complications, including fibrosis and cirrhosis [2]. The concomitant rise in metabolic syndrome-related conditions, including type 2 diabetes, dyslipidemia, and obesity, further underscores the urgency of addressing LD as a significant public health concern [3,17].

In the setting of increasing CRC incidence and mortality rates [7,8], our study reveals a compelling association between LD and CRC, urging healthcare professionals to consider LD as a potential risk factor during CRC screening. This underscores the need for heightened awareness and proactive screening strategies targeting individuals with LD to mitigate the burden of CRC and improve patient outcomes.

Strengths and limitations

Although our study sheds light on the association between LD and CRC, certain limitations warrant consideration. As this was a retrospective, cross-sectional analysis conducted at a single community hospital, the generalizability of our findings may be limited. The cross-sectional design prevents us from establishing a temporal relationship between LD and CRC, meaning we cannot determine whether LD precedes CRC or vice versa. This limitation underscores the need for longitudinal studies to establish causality. Additionally, potential confounders such as dietary habits, physical activity levels, and genetic predispositions were not controlled for, which could influence the observed associations.

Selection and information biases stemming from incomplete data and exclusion criteria further underscore the need for larger, multi-center studies with standardized methodologies to validate and extend our findings. Despite these limitations, our study's strengths include the rigorous confirmation of CRC and LD diagnoses through colonoscopy and histopathology, and a comprehensive analysis of a diverse set of clinical and demographic variables.

Future research

Future research should focus on longitudinal cohort studies to establish the temporal relationship between LD and CRC. Investigating the mechanistic pathways linking LD and CRC, such as inflammation, insulin resistance, and gut microbiota alterations, could provide deeper insights. Additionally, multi-center studies with larger and more diverse populations are needed to enhance the generalizability of the findings. Research should also explore whether the presence of MASLD should modify the threshold for CRC screening and the potential benefits of tailored screening protocols for individuals with LD [18].

## Conclusions

The data gathered in this study suggest that patients with a previously diagnosed liver disease (LD) face an increased risk of developing colorectal cancer (CRC) compared to those without LD. Additionally, the study indicates that women, Caucasians, and non-Hispanic populations show a stronger association between CRC and pre-existing LD. However, due to a limited number of participants, the study could not establish a significant link between LD and CRC in patients under 45 years. While it is premature to categorize LD as a definitive risk factor for CRC, our findings lend support to this hypothesis. This suggests a potential clinical implication for heightened vigilance and proactive management of patients with LD. Early identification and intervention in these high-risk groups could be crucial in mitigating the burden of CRC.

In summary, our findings underscore the importance of considering LD in the context of CRC risk. They highlight the need for healthcare professionals to integrate LD status into CRC screening protocols, especially for high-risk populations, to enhance early detection and improve patient prognosis.
